# Neoadjuvant and Adjuvant Immunotherapy in Resectable NSCLC

**DOI:** 10.3390/cancers16091619

**Published:** 2024-04-23

**Authors:** Evangelia Bogatsa, George Lazaridis, Chrysoula Stivanaki, Eleni Timotheadou

**Affiliations:** Department of Medical Oncology, Aristotle University of Thessaloniki, Papageorgiou Hospital, 56429 Thessaloniki, Greece; liana_ioanna@windowslive.com (E.B.); timotheadou@auth.gr (E.T.)

**Keywords:** non-small cell lung cancer, resectable, early, immunotherapy, immune-checkpoint inhibitors, adjuvant, neoadjuvant, perioperative

## Abstract

**Simple Summary:**

Non-small cell lung cancer (NSCLC), even in early stages, has been a fatal diagnosis and a leading death cause worldwide for decades. The aim of this review is to analyze the steps towards immunotherapy in the pre-, post-, and perioperative setting of resectable lung cancer while highlighting the challenges that have arisen and the future considerations to take into account in order to optimize the therapeutic results.

**Abstract:**

Non-small cell lung cancer, even when diagnosed in early stages, has been linked with poor survival rates and distant recurrence patterns. Novel therapeutic approaches harnessing the immune system have been implemented in early stages, following the designated steps of advanced NSCLC treatment strategies. Immune-checkpoint inhibitor (ICI) regimens as monotherapy, combinational, or alongside chemotherapy have been intensely investigated as adjuvant, neoadjuvant, and, more recently, perioperative therapeutic strategies, representing pivotal milestones in the evolution of early lung cancer management while holding great potential for the future. The subject of current ongoing research is optimizing treatment outcomes for patient subsets with different needs and identifying biomarkers that could be predictive of response while translating the trials’ endpoints to survival rates. The aim of this review is to discuss all current treatment options with the pros and cons of each, persistent challenges, and future perspectives on immunotherapy as illuminating the path to a new era for resectable NSCLC.

## 1. Introduction

Through the years, while tremendous progress has been made in all fields of oncology, only few things have been untouched by the passage of time. Early lung cancer is a radiant example, whose discouraging survival rates have remained irreversible for years. Surgery in early lung cancer, despite its definite approach, only seems to postpone the inevitable, with high recurrence rates, even up to 70% according to disease stage, while most of recurrences account for distant sites [1].

Attempts in integrating adjuvant cisplatin-based chemotherapy have yielded contradictory results for a decade or more, with some demonstrating a statistically significant advantage in overall survival (OS), while others have failed to validate it [2]. The Lung Adjuvant Cisplatin Evaluation (LACE) meta-analysis, a large pooled analysis of five trials (ALPI, ANITA, BLT, IATL, and JBR10) summing up 4584 patients in total, confirmed the absolute benefit of both OS and disease-free survival (DFS): 5.4% at 5 years and 5.8%, respectively [3].

The frame shifted later on towards neoadjuvant cisplatin-based therapy for resectable NSCLC patients. A meta-analysis of thirteen trials demonstrated that neoadjuvant chemotherapy is a statistically significant beneficial addition to surgery, improving overall survival in those patients, including the most challenging in terms of therapeutic strategy: stage III [4]. However, shortly thereafter, it became evident and validated via a large meta-analysis of Lim et al. that the DFS and OS benefit from chemotherapy was the same whether given in the adjuvant or neoadjuvant setting [5]. As a result, the landscape around early-stage NSCLC has remained static for years [6].

**Figure 1:**
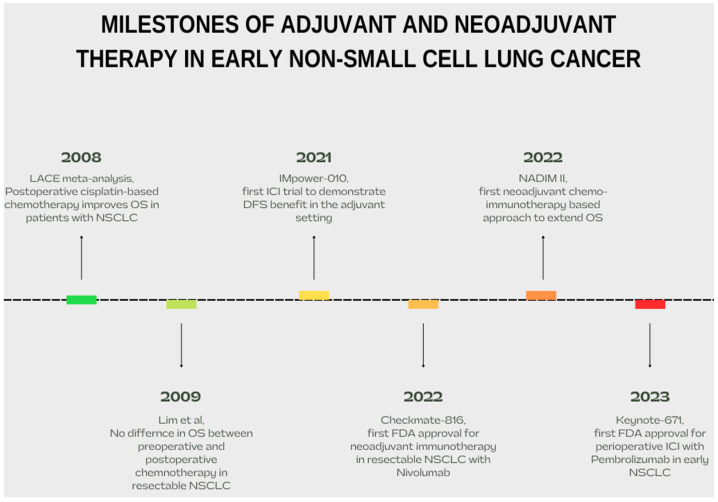
Milestones of adjuvant/neoadjuvant therapy in early NSCLC. Data from [3,5,9,10,11,12].

Immune checkpoint inhibitors have revolutionized the interface of lung cancer in advanced stages, with median OS extending over 20 months, when used as first-line treatment alongside platinum-based chemotherapy [7]. These remarkable outcomes have slowly seeded the ground for early-stage lung cancer immunotherapy trials, either combinational or as monotherapy, with promising results succeeding one another [Figure 1]. ICIs were first introduced postoperatively as adjuvant therapy after surgery. Soon, neoadjuvant immunotherapy trials emerged, leading to a much expected yet much needed merge: perioperative implementation of ICIs [8].

The purpose of this review is to elaborate on the rationale behind adjuvant and neoadjuvant immunotherapy in early-stage NSCLC, providing a brief discussion on the practice-changing clinical trials in both settings and identification of possible biomarkers that can prove to be useful regarding personalized therapy decision making as well as on the current unmet needs that could possibly be addressed in the future.

## 2. The Rationale behind Adjuvant and Neoadjuvant Immunotherapy

### 2.1. Adjuvant Setting

Surgical resection remains the cornerstone of early NSCLC management. Recurrence rates for patients who underwent primary surgery vary depending on disease stage, accounting for ~45% for stage IB while increasing to ~75% for stage III [3]. Irrespective of the modest outcomes, incorporation of adjuvant cisplatin-based chemotherapy regimens for patients who underwent complete surgical resection for early NSCLC was based on some reasonable arguments [Table 1]. Those arguments set the foundations for the first adjuvant ICI trials. First of all, the timing of the administration of adjuvant immunotherapy is a major advantage; it does not intervolve between or delay immediate surgical management and local disease control, nor does it jeopardize its curative intent with potential complications from systemic therapy, which may in some cases lead to surgery cancellation: ~20% of patients after neoadjuvant treatment do not make it to surgery due to systemic complications or disease progression that could occur in the meantime [13]. Additionally, flexibility in the initiation of adjuvant therapy in regards of time, providing for more time for recovery from surgery, is another significant asset, along with the potential for longer treatment duration without the urge to proceed to surgery [14,15]. In an effort to maximize the therapeutic effect of surgery, immunotherapy delivered postoperatively aims to eradicate micrometastatic disease. This is based on the premise that after surgical treatment, the neoplastic burden is substantially lower compared to the intact tumor load preoperatively and thus more susceptible to elimination [16]. Moreover, since surgery is proven to have a massive immunosuppressive impact on patients’ immune system, with postoperative stress and inflammation subsequently leading to release of inflammatory cytokines (IL-6, IL-8, IL-10, and TNF) and immunosuppressive cell subsets (T-regs, TAMs, and myeloid-derived suppressor cells), ICIs can serve as a stimulant to restore the postoperative impaired antitumor immunity [17]. Lastly, the “surgery-first” approach that is feasible with adjuvant administration of immunotherapy provides complete pathological staging of the disease as well as tissue-abundant samples for biomarker testing or other translational research that could be of use for tailored therapeutic strategies in the future. However, adjuvant immunotherapy entails some dangers that should be underlined. Treatment compliance rates are significantly lower within patients that underwent major surgery, sometimes reaching ~50% of patients that do not complete one year of adjuvant therapy, while the immunosuppressive environment post surgically could compromise immune responses to therapy [17,18].

### 2.2. Neoadjuvant Setting

Conversely, on the opposite side, we find neoadjuvant administration of immunotherapy [Table 1]. Steady templates of preclinical data from Liu et al. on breast cancer murine models, where the same immunotherapy regimen was administered pre- and postoperatively, showed that neoadjuvant treatment was superior to adjuvant in regards of survival when directly compared [19]. Trial data are not currently available for NSCLC patients; however, lessons from melanoma trials (Patel et al.) proved that a neoadjuvant/adjuvant approach of pembrolizumab in patients with resectable stage III–IV melanoma was more efficacious than an adjuvant-only approach [20]. The effectiveness of preoperative immune blockade therapy is enhanced by the intact tumor load available before resection, which serves as a huge neoantigen tank with the minimum possible tumor heterogeneity; as a result, robust immune responses and a broad T-cell expansion are initiated, a hypothesis first observed in the OpACIN study, where twenty patients with stage III melanoma treated with ICIs preoperatively presented with a plethora of different T-cell clones in the peripheral blood compared to patients that received the same regimen in the adjuvant setting [21]. Those immune responses are also characterized by durable outcomes and long-lasting activity, even after the initial lesion is surgically removed, which is effective against undetectable residual disease [22]. Furthermore, direct initiation of preoperative immunotherapy has been linked to early eradication of micrometastatic disease before seeding in distant organs through systemic circulation as well as the elimination of tumor cell shedding that may take place intraoperatively [23]. Equally important, pathologically assessed parameters after induction therapy are indicative of response and practically an in vivo evaluation of sensitivity to systemic therapy that can possibly guide and alter future treatment decisions in a personalized, patient-driven way [24]. Besides these, the patients’ performance status should be ideal before surgical intervention to undergo neoadjuvant treatment as well as complying optimally upon completion [25]. Last but not least, potential tumor downstaging before definitive surgery has been linked to more conservative surgical approaches and increases the percentage of R0 excisions [26].

### 2.3. Perioperative Setting

A combination of those traits came as no surprise soon after, as the first perioperative immunotherapy trials emerged [27]. Tumor downstaging and early eradication of micrometastatic spreading alongside postoperative control of residual tumor burden further strengthens surgical results and may establish long-term regression-free outcomes, a hypothesis highlighted by a plethora of ongoing perioperative trials that have built on this rationale [22]. 

## 3. Adjuvant Immunotherapy

The postoperative setting [Table 2] was the first to openly challenge the ability of ICIs in early-stage NSCLC patients. IMpower-010, a phase 3, randomized, open-label trial, tested the efficacy of atezolizumab in patients of stages II to IIIA (per TNM 7th) who had undergone upfront surgery. Around 1000 enrolled patients who had previously received at least one cycle of platinum-based adjuvant chemotherapy were randomized to either receive adjuvant atezolizumab for 1 year compared to best supportive care in the second arm of the study. IMpower-010 was the first randomized phase 3 study to succeed in demonstrating an apparent benefit in disease-free survival (DFS), the trial’s primary endpoint, with a postoperative ICI regimen. Median DFS was not reached (NR) in patients with positive PD-L1 expression ≥ 1% in the experimental arm versus 35.3 months in the best supportive care arm (HR 0.66; 95% Cl: 0.50, 0.88; *p* = 0.004); however, the results in the intention-to-treat (ITT) population did not cross the threshold of statistical significance. Interestingly, the trial did not exclude patients harboring driver mutations such as ALK rearrangements or EGFR mutations, who eventually did not benefit from adjuvant atezolizumab therapy, as shown in the trial’s subgroup analysis, alongside never smokers. In light of those results, the FDA granted approval of atezolizumab on 15 October 2021, a leap towards ICI incorporation in early NSCLC stages. EMA proposed approval of adjuvant atezolizumab only for the PD-L1 high-expressors subgroup, based on an exploratory analysis of IMpower-010; DFS was significantly higher in patients with the PD-L1 ≥ 50% subgroup compared to the 1–49% subgroup (HR 0.43 (95% CI 0.27–0.68) and 0.87 (95% CI 0.60–1.26), respectively). Lastly, data for overall survival in the ITT population remain immature to date; however, an interim analysis conducted at a cutoff of 25% of deaths demonstrated an evident trend towards it [9]. 

Another positive trial in the postoperative setting followed, testing the efficacy of adjuvant immunotherapy with pembrolizumab. KEYNOTE-091/PEARLS is a randomized phase 3 trial, during which 1177 participants were randomized to either receive pembrolizumab for up to 18 cycles or placebo, with primary endpoints DFS in overall population and in patients with PD-L1 TPS ≥ 50% and OS as a secondary endpoint. FDA approval was granted on 26 January 2023, with 18-month DFS rates at 73.4% with pembrolizumab versus 64.3% with placebo. Subgroup analyses indicated pembrolizumab was less effective for patients staged as IIIA, the ones who did not receive additionally adjuvant chemotherapy (optional and not mandatory in the trial design), and those with squamous histology [28].

While the two trials share much in common and are at first glance much alike, it is important to mention the key differences. The primary endpoints differ; DFS in PD-L1-positive patients was studied under the IMpower-010 trial, while DFS in the overall population was the primary endpoint in PEARLS. Secondly, the PD-L1 high-expressors (≥50%) subgroup demonstrated the biggest benefit in DFS (24%) with atezolizumab; however, interestingly, the same subgroup in PEARLS failed to show any statistically significant difference in DFS, a paradox attributed by many to the overperformance of the placebo group of the study. Lastly, different patient populations were included in each study; IMpower-010 did not exclude patients with EGFR driver mutations or ALK rearrangements, while it also recruited a slightly smaller-sized stage III patient population [9,28].

Results from trials assessing the use of adjuvant nivolumab and durvalumab (ANVIL and IFCT-1401, respectively) as monotherapy are still expected [32], while CANOPY-A, investigating adjuvant canakinumab, an IL-1βmonoclonal antibody known for its use in Still’s disease, failed to demonstrate benefit in DFS (median 35.0 months and 29.7 months with canakinumab versus placebo, respectively; HR 0.94 (95% CI 0.78–1.14) *p* = 0.258) [33].

## 4. Neoadjuvant Immunotherapy

CHECKMATE-159, a phase Ib/II, single arm, pilot study in 2018, was the first immunotherapy trial to deploy neoadjuvant nivolumab, administered for two cycles in patients with resectable NSCLC before surgery [10]. Major pathological response (MPR), defined as pathologically ≤ 10% of viable residual tumor cells, was observed in 45% of patients (9/20), alongside few reported adverse events and no negative correlation to surgical outcomes, while a recent 5-year update of the study demonstrated consistent findings, with an OS rate at 80% [10,34]. Phase 2 trials assessing neoadjuvant pembrolizumab and durvalumab (NEOMUN and IONESCO, respectively) were congruent in regard to results—although the latter was abruptly terminated due to postoperative complications—and prepared the immune milieu for the days that followed, and LCMC3, with two preoperative cycles of atezolizumab, also aligned in terms of MPR and pathologic complete response (pCR) [35,36].

Based on these encouraging results from phase 2 chemo-immunotherapy trials, CHECKMATE-816 enrolled patients with resectable, stage IB–IIIA NSCLC (TNM 7th) and showed that neoadjuvant nivolumab plus chemotherapy yielded impressive results when compared to platinum-based preoperative chemotherapy alone; pCR rates reached 24% compared to 2.2% of the standard-of-care chemotherapy regimen, and median event-free survival (EFS) rates were 31.6 months and 20.8 months (HR 0.63; 97.38% Cl 0.43–0.91; *p* = 0.005), respectively: a comparable safety profile in terms of grade 3–4 adverse events with no negative effect on surgical outcomes (successfully led to surgery in 83% versus 78% of patients). A post hoc analysis presented at the 2022 ESMO correlated pathological features with efficacy (pCR and EFS), navigating the dynamic towards the response-to-treatment biomarkers, a subject of current scholarly interest elaborated below [37]. Lastly, updates on 3-year EFS and OS rates were 72% vs. 47% and 85% vs. 66%, respectively [38].

An interesting update of CHECKMATE-816 EFS and OS results was presented at ESMO 2023 regarding the ipilimumab/nivolumab cohort of the study. It is apparent that within the first 12 months, some patients tend to perform worse, while initial treatment responders, in a longer follow-up, seem to maintain durable responses for an extended period of time until reaching an impressive plateau [39]. It is therefore crucial to identify beforehand this patient subset that does not perform well on a chemo-free regimen; a CHECKMATE 9LA approach—first-line nivolumab plus ipilimumab in combination with two cycles of platinum-based doublet chemotherapy—could be augmentative for those patients, especially the ones with negative-PD-L1 expression, but this is a question yet to be fully answered and should be further determined in the context of a clinical trial [40].

## 5. Perioperative Immunotherapy

**Figure 2:**
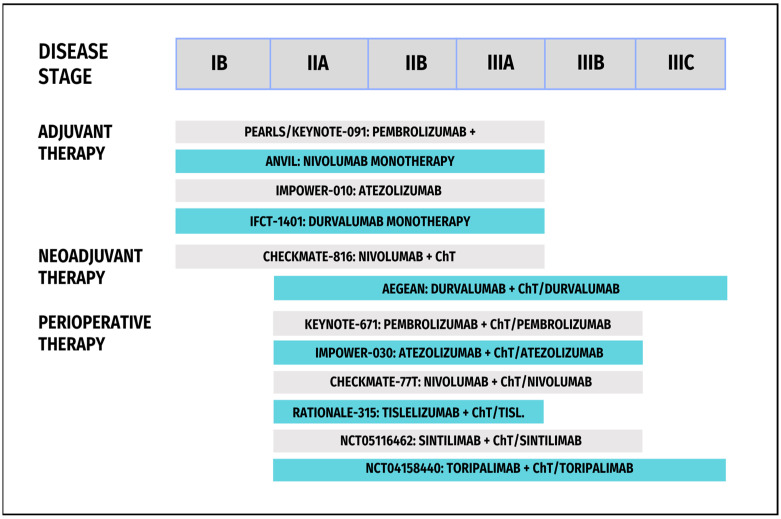
Major adjuvant/neoadjuvant immunotherapy trials per disease stage. Data from [9,10,12,28,29,42,43,44,45,46,47,48].

In the discourse of adjuvant versus neoadjuvant immunotherapy in resectable non-small cell lung cancer, a third option harboring great potential has emerged: perioperative immunotherapy [Figure 2]. Both adjuvant and neoadjuvant immunotherapy approaches represent successful treatment paradigms. With each alternative offering distinct advantages and disadvantages in different and heterogeneous patient groups, perioperative ICI schemes [Table 3], as aforementioned, seemed to have rationalized the complexity of this challenging entity [41].

Several studies were launched estimating the efficacy of either sequential or concurrent chemo-immunotherapy regimens; however, it was an open-label, single-arm, phase 2 trial that shook the very core of what has been known to date. The NADIM trial successfully recruited 46 patients with stage IIIA disease, who received three cycles of platinum-based chemotherapy plus nivolumab prior to surgery, which followed after 3 to 4 weeks after completion of the neoadjuvant treatment; they afterwards continued with adjuvant administration of nivolumab for one year. The results were quite impressive; the MPR rates were as high as 83%, with a subgroup of around 63% that achieved a complete pathological response—numbers never reported up to that point. The trial’s primary endpoint, the 2-year progression-free survival (PFS), was achieved for 77.1% of patients [37]. The synergistic combination of chemotherapy and immunotherapy was becoming rather evident, with preclinical data from Martin-Ruiz et al. demonstrating a significant difference in tumor regression rates in the arm that received immunotherapy plus cisplatin versus immunotherapy alone [49]. In light of NADIM’s results and building upon them, the NADIM II trial followed [50]. In this randomized, phase 2, open-label trial, 86 patients clinically staged IIIA–B, 35% of which presented with multi-stationed N2 disease, were randomly assigned to receive three cycles of doublet chemotherapy compared to nivolumab plus chemotherapy, followed by surgery and then adjuvant nivolumab for six months. Its primary endpoint, pCR in the ITT population, was 36.8% compared to 6.9% in the chemotherapy-only arm, which made NADIM II the first chemo-immunotherapy combinational trial to successfully correlate pCR with overall survival (12 months: 98.2% vs. 82.1%; 24 months: 84.7% vs. 63.4%) and progression-free survival benefit in a stage III-only population. This benefit was particularly observed in patients expressing PD-L1 who achieved complete pathological response [11]. Nonetheless, it has to be underlined that neither was designed to be evaluated as a primary endpoint of the study, a salient detail to bear in mind [51].

This pivotal trial served as proof of concept for five major trials that have built on the template of perioperative chemo-immunotherapy. The promising results from AEGEAN trial were first introduced during the annual AACR meeting of 2023. A total of 802 patients with stage IIA–IIIB disease were randomly assigned to either receive neoadjuvant durvalumab plus platinum-doublet chemotherapy or placebo plus chemotherapy for four cycles prior to surgery, followed afterwards by adjuvant durvalumab versus placebo for up to twelve cycles. A difference in achieved pCR rates was approximately 13.0% between the investigational and placebo arm without compromising surgery, as the number of patients that eventually underwent surgical treatment was numerically identical between the two arms (77.6% and 76.7%, respectively). EFS rates, the second primary endpoint of the trial, were 63.3% versus 52.4% in the experimental and placebo arm, respectively, at a 24-month follow up, while median EFS has not yet been reached for durvalumab compared to 25.9 months for the placebo. EFS and pCR benefit were found consistent irrespective of disease stage or PD-L1 expression [42].

The phase 3 NEOTORCH trial, assessing the efficacy of perioperative toripalimab, an anti PD-1 agent, recruited 404 patients staged as II–III, stratified by histology and PD-L1 status, who were randomized to either receive toripalimab or placebo in combination with chemotherapy for three cycles before surgery and one combinational cycle afterwards, followed by toripalimab or placebo monotherapy for thirteen cycles. Its primary endpoints were EFS and MPR rates. Overall, 77% of patients were of squamous histology, and N2 disease was present in 70% of patients, while 17% and 26% of patients, in the investigational and the placebo arm, respectively, did not eventually proceed with surgery. EFS rates at 24 months were 84.4% and 57% for the investigational and the placebo arm (HR 0.40), with a difference in rate of MPR at 40%. The EFS benefit was consistent across PD-L1 expression or histology. It must be noted, though, that the NEOTORCH was a trial conducted exclusively in a Chinese population, and the majority of patients had a diagnosis of squamous NSCLC [47].

Later on followed KEYNOTE-671, a phase 3 randomized trial of perioperative pembrolizumab, which published results of equivalent merit in the 2023 annual ASCO meeting. A total of 797 patients received either pembrolizumab or placebo alongside platinum-based chemotherapy for four cycles, followed by surgical treatment and postoperative continuation of the regimen for a maximum of thirteen cycles. Worthy of attention is the fact that patients harboring EGRF mutations or ALK translocations were not excluded from the trial. Event-free survival, the trial’s first primary endpoint, was found significant across all subgroups (NR versus 17.0 months; HR 0.58, 95% CI 0.46–0.72) regardless of histology, PD-L1 expression, or stage of disease [12]. Practice-changing results of KEYNOTE-671 were shared at the ESMO 2023 congress and further validated the efficacy of the previous interim analysis; perioperative pembrolizumab plus chemotherapy was the first ICI regimen to demonstrate a significant overall survival benefit (HR 0.72 [95% CI 0.56–0.93]; *p* = 0.00517), with OS rates at 36 months at 71.3% versus 64.0% for each arm and median OS not reached in the pembrolizumab arm versus 52.4 months in the placebo arm [42]. This led to the first FDA approval of immune blockade in the perioperative setting for early-stage, resectable NSCLC, while EMA’s Committee for Medicinal Products for Human Use (CHMP) also adopted a positive opinion for perioperative pembrolizumab on 22 February 2024, with the final decision expected within the first six months of the same year [52].

CHECKMATE-77T became the fourth randomized phase 3 trial to elucidate upon the findings of its predecessors, evaluating nivolumab plus chemotherapy versus placebo plus chemotherapy for four cycles before surgery in the perioperative setting of early NSCLC patients with the same disease profile. It was a positive trial with an improvement in EFS (2-year EFS rates at 70% for the nivolumab arm) and a hazard ratio equivalent to other regimens (HR: 0.58, *p* = 0.00025). The greatest EFS benefit amongst other subgroups was observed in patients having achieved pCR, patients who were eligible to continue with adjuvant nivolumab therapy, as well as patients who were staged as IIIA, which is a patient group linked with unfavorable prognosis and relatively high rates of micrometastatic spreading [43].

Lastly, the latest results from an interim analysis from RATIONALE-315 investigating the safety and efficacy of neoadjuvant/adjuvant tislelizumab (TIS), an anti PD-1 monoclonal antibody in combination with chemotherapy versus placebo and chemotherapy, with a dual primary endpoint of major pathologic response (MPR) and EFS, have been published. MPR and pCR rates were significantly improved (56.2% vs. 15.0% and 40.7% vs. 5.7%) in the TIS arm compared to placebo, in accordance to equally favorable EFS rates across all predefined subgroups (HR: 0.55, *p* = 0.0003). RATIONALE-315 is the fifth-in-a-row positive perioperative phase 3 study to share significant results, contributing to this remarkable progress for early NSCLC and, similarly to NEOTORCH, included Chinese-only population [44].

A recent meta-analysis involving seven major studies (Checkmate-816, TD-Foreknow, NADIM II, Keynote-671, Neotorch, Checkmate-77T, and AEGEAN) compared preoperative chemo-immunotherapy followed by surgery with preoperative chemotherapy alone. The benefit in EFS rates was evident in all included studies, with a total HR of 0.59 (95% CI: 0.52–0.66, *p* < 0.0001), while overall survival data, available in four out of seven studies (Checkmate-816, NADIM II, Keynote-671, Neotorch), were in favor of neoadjuvant immunotherapy approaches, with a significant reduction in the risk of death of 33% (HR: 0.67, 95% CI: 0.55–0.82, *p* < 0.0001). Subgroup analyses regarding performance status, age, disease stage, histology, the choice of platinum agent, as well as smoking history demonstrated a consistent benefit in EFS rates amongst all subgroups, while patients with high or intermediate PD-L1 expression were found to benefit the most when compared with non-expressors (PD-L1 < 1%), with HRs at 0.42, 0.56, and 0.75, respectively. Surgical and pathological outcomes were also available in all seven studies. There was a significant improvement in pCR rates with the addition of neoadjuvant immunotherapy to chemotherapy versus chemotherapy alone (21.8% vs. 3.8%, OR: 7.04, 95% CI: 5.23–9.47, *p* < 0.0001), alongside R0 resection rates (OR: 1.63, 95% CI: 1.24–2.14, *p* = 0.0005). A trend towards lower pneumonectomy rates was observed, however, without being statistically significant. Lastly, all-grade AEs were similar in both chemoimmunotherapy and chemotherapy groups (96.2% vs. 95.8%, respectively), although a noted increase in grade 3–4 AEs was observed in patients who received chemoimmunotherapy (45.1% vs. 41.7% for chemotherapy alone) and must therefore be mentioned (Guven et al. [53]).

## 6. Surgical Outcomes

As mentioned above, survival in NSCLC varies according to disease stage, with optimal R0 resections as the backbone of treatment in early stages, and is vastly correlated with overall survival prolongation [54]. Perioperative strategies with ICIs have offered a holistic approach to early NSCLC. While adjuvant approaches are supplementary to surgery and enhance its outcome, preoperative administration of (chemo)immunotherapy has raised concerns regarding delays in surgical programming, morbidity, or treatment-related toxicities. Technical difficulties in patients under neoadjuvant treatment may also arise, compromising intraoperative parameters [55].

However, in none of the perioperative trials mentioned did the surgical cancellation rate exceed ~20%—a rate that dropped below half in neoadjuvant trials with ICIs alone and no chemotherapy compartment [Table 4]. Moreover, this percentage included cases of disease progression, which, by definition, rendered the tumor inoperable [56]. For instance, in CHECKMATE-816, numerically more patients underwent surgery compared to the placebo arm [37]. Additionally, neoadjuvant/perioperative approaches shift the dynamic towards significant tumor downstaging and thus more conservative surgical resections—more lobectomies than pneumonectomies and shorter operation times [57]. This approach opposes, though, the very dogma of lung cancer surgery, in which the extent of resection should be identical to the disease extent before neoadjuvant therapy, regardless of regression rate. So, questions arise as to whether less radical surgical approaches are actually in favor of patients, or on the other hand, it may be time to redefine current standards of care in the era of immunotherapy [58]. Defining resectability is another argument upon which to elaborate; post-neoadjuvant immunotherapy reassessment could be feasible, but such approaches should be studied in a clinical trial context [59].

Last but not least, omitting surgery in selected patients with exceptional response to neoadjuvant therapy, for example, patients with pCR or undetectable minimal residual disease post surgery, has not been studied to date, with only limited preliminary data available [19].

## 7. Biomarkers and Unmet Needs

### 7.1. Major Pathologic Response (MPR), Pathologic Complete Response (pCR), and Other Endpoints

Pathologic definitions such as MPR and pCR and their correlation with parameters of survival will probably shed more light on whether higher rates of pathologically assessed regression translate in prolonged survival. In order to achieve that, it is imperative that both pre- and post-therapy specimens be analyzed in order to identify those parameters that will operate as surrogate biomarkers to further optimize therapeutic outcomes [36].

Pathologically complete response rates were strongly correlated to 5-year survival rates in early chemotherapy-only studies by [60] (80% vs. 56% without pCR, *p* < 0.01). EFS and DFS are also widely studied as markers of surrogacy for overall survival. In early NSCLC chemotherapy trials, it was shown that 3-year DFS was sufficient to accurately predict a 5-year OS trend; however, extrapolating these results to immunotherapy trials has several limitations. To date, OS remains the most valid endpoint, although early assessment of results and treatment effects through validated surrogate markers is a direction to work towards [61].

After the NADIM II results of pCR and OS correlation, as mentioned above, the first to elaborate on the matter was a post hoc analysis of CHECKMATE-816 and neoadjuvant nivolumab, in which EFS was assessed in parallel with residual viable tumor (RVT) rates and found to be prolonged in the specimens of patients whose RVT was closer to zero; pCR rates in accordance to EFS were also analyzed and found strongly correlated (HR 0.13) [37]. Likewise, CHECKMATE-77T published the results of an exploratory analysis of EFS based on pCR and MPR status; these results also confirmed that EFS is higher in patients achieving pathologically complete response [43]. In the same pattern, an exploratory analysis of KEYNOTE-671 evaluating EFS rates by MPR rates (HR: 0.54 with MPR, HR: 0.73 without MPR) also shared favorable results; patients achieving major or complete pathologic responses post neoadjuvant therapy have greater likelihood for longer EFS intervals. So, it is evident that in-depth analysis of these histopathology features and direct correlation with survival parameters may be augmentative to personalizing treatment approaches as questions about the role of adjuvant after neoadjuvant treatment arise between under- and overtreatment for each individual [62].

### 7.2. PD-L1 Expression

Around 40% of early NSCLC patients seem to gain no benefit from chemo-immunotherapy regimens, highlighting the unmet need for customized patient care [50]. Amongst other similar parameters, such as tumor mutational burden (TMB), CD8+ invasion rates, and absence of immunosuppressive cell subsets, PD-L1 expression has emerged as the most clinically significant biomarker to assess response to immunotherapy [16].

The three-year efficacy outcomes of CHECKMATE-816 support PD-L1 as a biomarker for EFS; the assessed pCR, EFS, and OS were substantially higher in patients with PD-L1 expression ≥ 1%. Another example in this setting accounts for IMpower-010 and atezolizumab, whose subgroup analysis demonstrated DFS benefit for the PD-L1-positive population and no benefit in patients with negative PD-L1 expression [9]. Early OS analysis of the same trial for PD-L1 ≥ 1% has not yet reached statistical significance; however, overall survival for PD-L1 high expressors is clearly in benefit, with HR 0.42 [37]. So, it is widely hypothesized that PD-L1 expression could serve as a predictive biomarker in neoadjuvant/perioperative immunotherapy trials [63]. However, on the other hand, objective responses were observed even in PD-L1 non-expressors; KEYNOTE-671 demonstrated consistent benefit across PD-L1 expression, although it was remarkably lower in PD-L1 non-expressors compared to patients with PD-L1 ≥ 1% (HR: 0.77 vs. HR: 0.51) [12], while in PEARLS, as aforementioned, the PD-L1 >50% subgroup did not perform accordingly [Table 5] [28].

### 7.3. ct-DNA and Minimal Residual Disease (MRD)

Neoadjuvant/adjuvant incorporation of ICIs is claiming its rightful position in early lung cancer management. Although results are in general remarkable, as more data become mature and ready to publish day by day, the more evident the need for personalized approaches becomes. The scale must balance between tailored treatment and overtreatment; for instance, there are patients who receive the adjuvant component of the perioperative scheme but, in fact, gain no benefit. In addition, the vast majority of adverse events occurs postoperatively, during the adjuvant period, which is longer by definition and includes events that could potentially result in chronic defects or even treatment-related deaths. It is, therefore, crucial to identify those patient subgroups that gain nothing but potential toxicity by completing a full perioperative schedule [64]. Similarly, the number of cycles and the duration of adjuvant treatment after surgery is a debate of interest these days. The financial parameter, lastly, is not something to neglect either [65]. Other than that, although indirect comparison of trials is out of the question due to the risks of comparing different populations, selection criteria, and stage distribution, a similar impact in OS trend is observed across neoadjuvant and perioperative approaches (CHECKMATE-816—HR:0.62, *p* = 0.0124; KEYNOTE-671—HR: 0.72, *p* = 0.00517; NEOTORCH—HR:0.62, *p* = 0.00502; RATIONALE-315—HR: 0.62, *p* = 0.0193); therefore, to date, it is rather impossible to define which strategy is superior [10,11,42,52].

Alongside MPR/pCR, as discussed above, detecting MRD via circulating-tumor DNA is under evaluation as a surrogate marker to assess treatment response and identify those subgroups in higher risks of relapse, which tend to benefit more from adjuvant treatment completion. Under the LCMC3 trial and atezolizumab, half of patients with MPR had ctDNA clearance, which correlated with improved DFS. After that, an exploratory analysis of the AEGEAN trial of perioperative durvalumab published a thorough ctDNA analysis, studying the parameters of ctDNA levels and their fluctuations in accordance with treatment phase as well as ctDNA clearance rate by MPR/pCR rates. Variant-allele frequency (VAF) and ctDNA clearance rates harbored predictive utility; patients without ctDNA clearance were unlikely to achieve major pathologic responses, whereas rapid clearance rates, observed predominantly in the durvalumab arm, were strongly associated with higher pCR rates. Another example of ctDNA analysis was conducted in IMpower-010 and adjuvant atezolizumab. DFS rates were clearly superior for the patient subgroup that achieved ctDNA clearance compared to the detectable ctDNA subgroup in both atezolizumab and the best supportive care arms, which establishes ctDNA detection as a strong prognostic marker for DFS, although it is of no predictive value at this point. Therefore, ctDNA could represent a surrogate marker for early MRD detection, identifying patients that may relapse early after treatment, in order to intensify the therapeutic strategy as well as optimally design further treatment plans [9,66].

## 8. Other Novel Immunotherapy Approaches

Early lung cancer treatment is, at the same time, directed towards other novel forms of immunotherapy, cancer vaccines, and cell therapies. Cancer vaccines harbor the ability to induce specific T-cell responses against tumor antigens, although with limited clinical activity to date. The large phase 3 MAGRIT trial, investigating the efficacy of a shared antigen vaccine, MAGE-A3, as adjuvant therapy for patients with resected NSCLC who tested positive for MAGE-A3, yielded with negative results regarding DFS, its primary endpoint, and OS, with several other negative trials following [67]. Nowadays, cancer vaccines move towards a more personalized approach and patient-driven path compared to the shared antigen strategy of older attempts. Recently published results from KEYNOTE-942, a phase 2b study with patients with resected stage IIB–IV high-risk melanoma, presented encouraging data. Administration of a personalized neoantigen mRNA-based vaccine (mRNA-4157) in those patients along with pembrolizumab compared to pembrolizumab monotherapy demonstrated clinical utility and extended recurrence-free survival [68]. Of similar principle, after the promising INTerpath-001 in melanoma, patients with resected stage II–IIIB NSCLC were recruited to the currently ongoing phase 3 trial INTerpath-002; these patients were randomized, after adjuvant chemotherapy, to either receive pembrolizumab plus an individualized neoantigen mRNA vaccine (mRNA-4157) every three weeks for up to nine doses or pembrolizumab plus placebo [69]. As with any innovational approach, questions arise as to when cancer vaccines perform better: in adjuvant or advanced setting or combined with or without other immunotherapy agents. What is certain is that personalized cancer vaccines have to prove efficacious in metastatic setting before being brought forward in earlier stages of NSCLC.

On the other hand, adoptive T-cell therapies move in different strategic paths, incorporating TILs, CAR T cells, and TCR T cells [70]. TILs, in particular, are autologous cancer cells derived from the patient’s tumor during a complex but one-time procedure. An adoptive therapy with TILs was recently granted accelerated approval in patients with metastatic melanoma. Lifiluecel was administered in patients pretreated with anti-PD-1 blockade or BRAFi +/− MEKi, with many of them responding early and maintaining a prolonged duration of response for over a year or two, with, however, a concerning adverse events profile [71]. Similar strategies are being investigated in advanced and refractory NSCLC and are highly awaited.

## 9. Quality of Life (QoL) and Health-Related Parameters

Quality of life parameters is an emerging topic of growing interest in immunotherapy trials. Compared to standard chemotherapy regimens and their known adverse events, ICI schemes are generally well tolerated, even in patient subsets with worsened performance status. When it comes to lung cancer, balancing the quality of life scale and treatment efficacy in the metastatic setting is somewhat easier; quality of life is of great importance since there is not, by definition, any curative intent. Therefore, it is crucial to minimize both disease-related symptoms as well as treatment-induced side effects. And this has been the case, with several analyses and meta-analyses in metastatic NSCLC focusing on the matter and evaluating quality of life on a subjective scale through questionnaires and various parameters as surrogates: emotional, physical, social, and many others. Immunotherapy is both very well tolerated and a major contribution to quality of life among the patients who receive it [72,73,74].

Incorporation of ICIs in the early stages of lung cancer, where survival rates are substantially higher, highlights the importance of assessing such parameters in these patients and should not be understated. In a setting where the ultimate goal is to cure, minimizing chronic toxicities and long-lasting side effects is equally as important as treatment efficacy. Most of the aforementioned trials have included health-related quality of life (HRQoL) parameters as exploratory endpoints. For example, in Checkmate-816, no detrimental effect was reported after adding immunotherapy to chemotherapy, with most patients tolerating the regimen well. HRQoL data are also awaited from other immunotherapy trials, especially in the postsurgical period, that better assess the effect of adjuvant immunotherapy on the quality of life, as longer follow-up data become available. Clearly, all future research should entail health-related symptoms and quality of life measures, defined by both patients and physicians, to capture the holistic effects of this landscape-reshaping approach on patients [75].

## 10. Conclusions and Future Challenges

Lung cancer is one of the leading causes of cancer death worldwide, with recurrence rates remaining high even in the early stages of NSCLC. At the forefront of this therapeutic frontier, immunotherapy has, up to a certain point, rationalized this complex issue, presenting impressive results. However, a myriad of considerations must be taken into account to discern the best therapeutic approach, with each avenue offering distinctive advantages as well as challenges; from adjuvant immunotherapies aiming to eradicate microscopic remnants against the specter of recurrence to neoadjuvant ICI approaches, where harnessing the body’s innate defense mechanisms from the beginning orchestrates an immune response against malignant cells, perioperative immunotherapy has succeeded in merging the synergistic interplay of the two approaches, possibly rising as the new standard of care for this patient population. Based on the available data, it has become evident that patients that perform better with immunotherapy are the ones who achieve MPR/pCR, those with undetectable ctDNA levels post treatment, and the subset of high PD-L1 expression. To further maximize therapeutic outcomes, effective treatment plans are necessary to completely eradicate micrometastatic disease and improve survival rates, tumor downsizing, and optimization of surgical parameters, along with early surrogate markers to intensify adjuvant treatment or modify the therapeutic strategy accordingly, minimizing overtreatment and toxicity. At this point, detailed reverse-translational research on blood and tumor biospecimens to further determine response, resistance, or new therapeutic agents is the means to work towards it.

However, despite all emerging data, it is still challenging to select patients to receive neoadjuvant therapy before surgery and patients to undergo upfront surgical intervention followed by adjuvant therapy. The greatest benefit of the neoadjuvant strategy seems to be obtained by stage III patients, who are by definition in higher risk of recurrence and have a worse prognosis, although, in general, the results seem independent of stage in most trials; an exception to this paradigm was shown in KEYNOTE-671, whose subgroup analysis demonstrated better EFS rates in stage II patients than in stage III patients. The role of PD-L1 as a predictive biomarker has already been analyzed above, but its patient selection abilities are still questionable. Checkmate-159 was the first trial to demonstrate a positive correlation between pretreatment PD-L1 levels and favorable outcomes, with several similar results from NADIM, NADIM II, and LCMC3 following it. It is unquestionably a useful marker of clinical benefit; however, selecting or excluding patients based on PD-L1 expression alone could have detrimental consequences, as patients with negative expression below 1% have achieved pCR after neoadjuvant ICI treatment [75]. Lastly, research on molecular biomarkers and signatures is currently under scrutiny; an interesting analysis was concluded by Checkmate-816, for instance, where a four-gene (CD8A, STAT1, LAG3, and CD274) inflammatory signature score was analyzed by pCR and MPR. A high or low inflammatory signature score was also correlated with EFS, with rates at 36 months of 70% and 50%, respectively, for high and low scores for the immunotherapy arm compared to almost identical numerical rates for the chemotherapy arm (Awad et al., ESMO 2023 [39]). Such analyses harbor great potential regarding decision making in the future, although currently only at the very early stage, and nonetheless, they require more thorough research to be translated and incorporated into clinical practice [75,76,77].

Another hurdle to overcome is determining the optimal neoadjuvant treatment duration; while a three-cycle only neoadjuvant ICI treatment with no adjuvant component, as in Checkmate-816, could stand as a financially cost-effective approach, intensifying the algorithm for patients who failed to achieve pCR arose as a matter of debate. The first to elaborate on the matter was the phase 2 NeoSCORE trial, in which two cycles of neoadjuvant sintilimab plus chemotherapy was compared to three cycles, with a numerical benefit of MPR at 14.5%; however, it was not statistically significant. Results from the phase 3 NeoSCORE trial in resectable squamous cell carcinoma, evaluating the difference between three and four cycles of neoadjuvant sintilimab plus chemotherapy, are currently awaited [78,79]. Most neoadjuvant/perioperative trials, though, suggest 3–4 cycles of immunotherapy prior to surgery, with, as aforementioned, similar hazard ratios to the extent that this comparison is feasible. Balancing between benefit and toxicity is critical, and a retrospective study tried to answer this question, analyzing data from 251 patients who had received either 2 or 3–4 cycles of preoperative immunotherapy. Findings of numerical benefit regarding MPR rates, surgical safety, and more treatment-related adverse events were observed with more preoperative cycles, although such results must be further investigated in a prospective context to safely draw reliable conclusions [80].

Accordingly, the optimal duration of the adjuvant component of perioperative immunotherapy or even discontinuation is also an unsolved problem at the moment. A trending benefit of survival was observed among patients with R0 resection in the NADIM II trial who completed adjuvant immunotherapy after surgery compared to those who did not complete the adjuvant ICI treatment part. Similarly, in KEYNOTE-671, an EFS benefit was recorded for patients who received adjuvant immunotherapy compared to the patients who did not. However, it is evident that real-world patients experience more AEs compared to trials’ populations. It is also evident that not all patients gain the same benefit from the same treatment. The first question to arise, then, is whether patients achieving pCR should receive adjuvant therapy at all; the prognosis after pathologically complete response is excellent, and a possibly unnecessary intervention with adjuvant ICI makes these patients prone to potentially chronic defects and long-lasting immune-related adverse events. Given that the intent of all these approaches is curative, it is of great importance to minimize potential risks of lifetime consequences in patients that could otherwise be free of disease, and since most of AEs occur in the adjuvant setting of the perioperative schemes, mainly due to its longer duration, it is crucial to tailor the therapeutic schedule according to the patient in question. For instance, although underrepresented in many trials, patients with stage II disease have better OS rates [75]. Additionally, as mentioned above, patients who achieve pathologically complete response also achieve better EFS and OS rates. So, questions arise as to whether every patient benefits from receiving the full schedule of the adjuvant component of perioperative immunotherapy. De-escalation conclusions have been sought in a different setting, definitive chemo-radiotherapy; they are, however, worthy of mentioning. A retrospective analysis of consolidation durvalumab after concurrent chemoradiotherapy showed that similar PFS rates were recorded at 9 and 12 months of consolidation immunotherapy, but the results were inferior for patients who received durvalumab for 6 months. Although it may serve as an indirect correlation of treatment duration and efficacy, such analyses can only point towards the right direction, and it once again shows that prospective data are necessary to further validate such assumptions [81]. CtDNA dynamics could also prove handy in resolving this issue, with recently proposed criteria of response evaluation (LB-RECIST), although, again, prospective data are lacking [82]. Short follow-up times and immature survival data are inadequate, at least at the moment, to safely determine the duration of perioperative immunotherapy, especially the adjuvant part. A second question that remains unanswered is whether patients that fail to achieve pCR or MPR do actually benefit from continuing on the same immunotherapy protocol and whether treatment escalation attempts should be made, perhaps with the addition of another ICI agent—CTLA-4, LAG-3, etc.—or ADCs to achieve better therapeutic outcomes. To date, there is no strong evidentiary support that response to neoadjuvant treatment should further guide adjuvant treatment decisions [53,75,83,84].

In the same context, another important issue to address is the feasibility of surgery in patients with conventionally unresectable disease, pushing the boundaries further. Many studies have involved patients with IIIB disease: 35% of patients in NADIM II presented with multistational N2 disease, 25% of patients in each arm of the AEGEAN study had IIIB disease, and half of patients in each arm had N2 disease, some of which was multistational. Around 70% of NEOTORCH patients had N2 disease: 30% in multiple stations, and 15% out of 45% of N2 patients in Checkmate-77T were also multistational. A subgroup analysis of the latter demonstrated benefit among this patient population, with HR at 0.43. These data, alongside pending results from ongoing studies, could redefine resectability criteria and include more patients as feasible for surgery [53,75,83].

Yet, as with any new endeavor, uncertainties and challenges regarding timing, optimal patient selection, treatment duration, and tumor biology have surfaced, highlighting the need for a more precise, patient-tailored care provision. Most population data come from subgroup analyses, and patients were not stratified beforehand accordingly, so drawing definite conclusions is rather impossible at this point. The optimal treatment route for patients of stage II or III; patients with/without MPR or pCR post neoadjuvant therapy; patients with high, intermediate, or low PD-L1 expression; or other characteristics does not yet exist. Clinicians must personalize therapeutic plans according to patients until more data become available. It is a multifactorial matter of thoughtful analysis to identify those patient populations with the greatest benefits from immunotherapy while mitigating adverse events to the minimum possible percentage. Nevertheless, immune blockade has unveiled new opportunities to help reshape early-stage NSCLC management.

## Figures and Tables

**Table 1:** Advantages and disadvantages of pre- and postoperative immunotherapy strategies (as discussed in the main text).

Adjuvant Immunotherapy		Neoadjuvant Immunotherapy	
PROS	CONS	PROS	CONS
No delay/cancellation of surgical approach	Poor treatment compliance	High neoantigen load initiates robust immune responses	Treatment delays/cancellation/disease progression; ~20% do not undergo surgery
Low tumor burden—eradication of residual micrometastatic disease	Highly immunosuppressive environment post surgery—less antigenicity	Broad T-cell priming	Preoperative tissue sampling is mandatory
Flexibility in time of therapy and longer treatment duration	Risk for chronic immune-related AEs	Early eradication of micrometastatic spreading	Not all patients benefit from neoadjuvant therapy
Restores impaired anti-tumor immunity after surgery	Financial toxicity	Tumor downstaging—more conservative approaches and R0 excisions	
Information of postoperative specimen before treatment	Overtreatment concerns	Better compliance—optimal timing to start therapy	
		Pathologically assessed parameters—surrogate biomarkers	

**Table 2:** Adjuvant immunotherapy trials and their characteristics. Data from [9,28,29,30,31].

Adjuvant	Stage	Drug	Regimen	Sample Size	Phase	Primary Endpoint	HR	FDA Approval
IMPOWER-010	IB–IIIA	ATEZOLIZUMAB	ATEZO + CHT/PLACEBO	1280	3	DFS	0.81	10/2022
PEARLS/KEYNOTE-091	IB–IIIA	PEMBROLIZUMAB	PEMBRO/PLACEBO	1177	3	DFS	0.76	01/2023
ANVIL	IB–IIIA	NIVOLUMAB	NIVO/PLACEBO	903	3	DFS, OS	-	-
MERMAID 1	II–III	DURVALUMAB	DURVA + CHT/PLACEBO	86	3	DFS in ctDNA+	-	-
MERMAID 2	II–III	DURVALUMAB	DURVA/PLACEBO	284	3	DFS	-	-

**Table 3:** Perioperative/neoadjuvant immunotherapy trials and their characteristics. Data from [10,12,42,43,44,47,48].

PERIOPERATIVE/NEOADJUVANT	STAGE	DRUG	REGIMEN	SAMPLE SIZE	PHASE	PRIMARY ENDPOINT	HR	FDA APPROVAL
KEYNOTE-671	IIB–IIIA	PEMBROLIZUMAB	PEMBRO + CHT-SURGERY-PEMBRO	786	3	EFS, OS	0.59	10/2023
CHECKMATE-77T	II–IIIB	NIVOLUAB	NIVO + CHT-SURGERY-NIVO	452	3	EFS	0.58	-
IMPOWER-030	II–IIIB	ATEZOLIZUMAB	ATEZO + CHT-SURGERY-ATEZO	450	3	MPR, EFS	-	-
RATIONALE-315	II–IIIA	TISLELIZUMAB	TIS + CHT-SURGERY-TIS	380	3	MPR, EFS	0.56	-
NEOTORCH	IIIA	TORIPALIMAB	TORI + CHT- SURGERY-TORI	406	3	MPR, EFS	0.40	-
AEGEAN	IIA–IIIB	DURVALUMAB	DURVA + CHT- SURGERY-DURVA	800	3	MPR	0.68	-
CHECKMATE-816	IB–IIIA	NIVOLUMAB	NIVO + CHT/CHT	350	3	EFS, pCR	0.63	03/2022

**Table 4:** Surgical parameters and outcomes of major pre-/perioperative immunotherapy trials. Data from [10,12,42,43,47].

SURGICAL OUTCOMES	KEYNOTE-671 PEMBRO vs. PLACEBO	CHECKMATE-77T NIVO vs. PLACEBO	NEOTORCH TORI vs. PLACEBO	AEGEAN DURVA vs. PLACEBO	CHECKMATE-816 NIVO vs. PLACEBO
RESECTED R0 RESECTION R1/R2 RESECTION	82.1 vs. 79.4 92.0 vs. 84.2 6.4 vs. 11.3	78.0 vs. 77.0 89.0 vs. 90.0 11.0 vs. 10.0	82.2 vs. 73.3 95.8 vs. 92.6 4.2 vs. 7.4	80.6 vs. 80.7 94.7 vs. 91.3 4.9 vs. 8.4	83.2 vs. 75.4 83.2 vs. 77.8 16.8 vs. 22.2
SURGICAL PROCEDURE LOBECTOMY PNEUMONECTOMY OTHER	78.8 vs. 75.1 11.4 vs. 12.3 9.8 vs. 12.6	80.0 vs. 72.0 9.0 vs. 14.0 11.0 vs. 14.0	80.7 vs. 83.1 9.0 vs. 9.5 10.3 vs. 7.4	88.1 vs. 85.4 9.2 vs. 9.6 2.7 vs. 5.0	77.0 vs. 61.0 17.0 vs. 25.0 19.0 vs. 26.0
ADJUVANT THERAPY	73.2 vs. 66.9	62.0 vs. 66.0	71.3 vs. 64.9	65.8 vs. 63.4	-

**Table 5:** Outcomes per PD-L1 expression in major pre-/perioperative immunotherapy trials. Data from [10,12,42,43,44,47].

	PD-L1 < 1%		PD-L1 1–49%		PD-L1 ≥ 50%	
TRIAL	pCR (%)	HR (95%CI) EFS	pCR (%)	HR (95%CI) EFS	pCR (%)	HR (95%CI) EFS
CHECKMATE-816	16.7 vs. 2.6	0.85 (0.54–1.32)	23.5 vs. 0	0.58 (0.30–1.12)	44.7 vs. 4.8	0.24 (0.10–0.61)
KEYNOTE-671	-	0.77 (0.55–1.07)	-	0.51 (0.34–0.75)	-	0.42 (0.28–0.65)
AEGEAN	9.0 vs. 3.2	0.76 (0.49–1.17)	16.3 vs. 4.9	0.70 (0.46–1.05)	27.5 vs. 4.7	0.60 (0.35–1.01)
NEOTORCH	-	0.59 (0.32–1.03)	-	0.31 (0.17–0.55)	-	0.31 (0.15–0.61)
CHECKMATE-77T	12.9 vs. 4.3	0.73 (0.47–1.15)	26.5 vs. 3.9	0.76 (0.46–1.25)	51.1 vs. 5.8	0.26 (0.12–0.55)
RATIONALE-315	-	0.80 (0.47–1.38)	-	0.34 (0.17–0.66)	-	0.71 (0.38–1.34)
